# Conditional inactivation of Akt three isoforms causes tau hyperphosphorylation in the brain

**DOI:** 10.1186/s13024-015-0030-y

**Published:** 2015-07-31

**Authors:** Long Wang, Shanshan Cheng, Zhenyu Yin, Congyu Xu, Shuangshuang Lu, Jinxing Hou, Tingting Yu, Xiaolei Zhu, Xiaoyan Zou, Ying Peng, Yun Xu, Zhongzhou Yang, Guiquan Chen

**Affiliations:** Model Animal Research Center, MOE Key Laboratory of Model Animal for Disease Study, Nanjing University, 12 Xuefu Avenue, Nanjing, Jiangsu Province 210061 China; Department of Geriatric, Nanjing Drum Tower Hospital, Nanjing University Medical School, 321 Zhongshan Avenue, Nanjing, Jiangsu Province 210008 China; Institute of Materia Medica, Chinese Academy of Medical Sciences & Peking Union Medical College, Xuanwu District, Beijing, 100050 China; Department of Neurology, Nanjing Drum Tower Hospital, Nanjing University Medical School, 321 Zhongshan Avenue, Nanjing, Jiangsu Province 210008 China

**Keywords:** Akt, Tau phosphorylation, Tau kinases, GSK3 and PKA

## Abstract

**Background:**

Tau hyperphosphorylation plays a critical role in neurodegenerative diseases [EMBO Mol Med. 6:1142-60, 2014; Annu Rev Neurosci. 24:1121-59, 2001]. Recent evidence has shown that Akt is down-regulated in AD [J Pathol. 225:54-62, 2011]. However, it remained unknown which pathological process, e.g. tau pathology or neuron death, Akt may contribute to. In this study, Cre-loxP technique was employed to generate a viable Akt three isoforms conditional knockout (*Akt* cTKO) mouse in which total Akt levels were dramatically reduced in the adult brain.

**Results:**

Significantly increased levels of tau phosphorylated (p-tau) at various sites were observed in *Akt* cTKO mice as compared to age-matched littermate controls. Increased levels for phosphorylated GSK3α and phosphorylated PKA substrates were detected in *Akt* cTKO brains. In contrast, no significant changes on p-tau levels were found in *Akt1*^−/−^, *Akt2*^−/−^ or *Akt3*^−/−^ mice.

**Conclusions:**

Akt may regulate tau phosphorylation in the adult brain by affecting activities for PKA and GSK3α.

**Electronic supplementary material:**

The online version of this article (doi:10.1186/s13024-015-0030-y) contains supplementary material, which is available to authorized users.

## Findings

Mice with global deletion of Akt three isoforms (*Akt* TKO or *Akt1*^*−/−*^*;Akt2*^*−/−*^*;Akt3*^*−/−*^) were early embryonic lethal [1], precluding the possibility to study its *in vivo* function. To obtain viable *Akt* conditional TKO (cTKO) mouse, we generated floxed *Akt1* (Additional file 1: Figure S1A). After several steps of crossing, *Akt1*^*f/f*^*;Akt2*^*−/−*^*;Akt3*^*−/−*^*;CAG-CreER* mice were obtained and became *Akt* cTKO after the treatment with tamoxifen. To examine Cre-mediated recombination efficiency in the brain, biochemical analyses were conducted. Dramatic reduction on levels for total Akt (t-Akt) was observed in the brain of cTKO mice (Additional file 1: Figure S1B). Levels of p-Akt^Ser473^, an activated form of Akt, were markedly decreased in *Akt* cTKO (Additional file 1: Figure S1B). Immunohistochemistry (IHC) results showed very weak immuno-reactivity of t-Akt in the brain of *Akt* cTKO mice (Additional file 1: Figure S1C-d-f, a-c for controls).

**Fig. 1:**
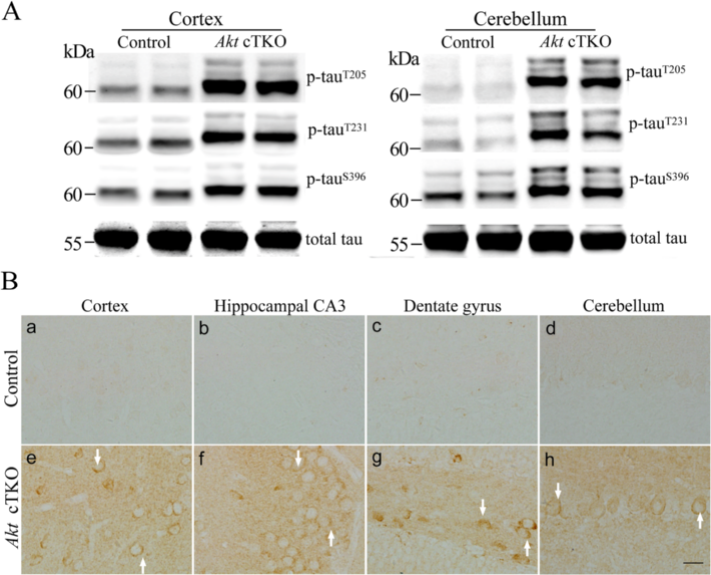
Tau hyperphosphorylation in the brain of *Akt* cTKO mice. **a** Western blotting of p-tau using cortical and cerebellar samples. Antibodies against tau phosphorylated at the Thr205, Thr231 and Ser396 epitopes were used. The migration of three p-tau bands (60 kDa, 64 kDa and 68 kDa) in SDS gels was slower in *Akt* cTKO (*Akt1* ^*f/f*^ *;Akt2* ^*−/−*^ *;Akt3* ^*−/−*^ *;CAG-CreER* with tamoxifen treatment) than in control mice (*Akt1* ^*f/f*^ *;Akt2* ^*+/+*^ *;Akt3* ^*+/+*^ and *Akt1* ^*f/+*^ *;Akt2* ^*+/+*^ *;Akt3* ^*+/+*^). Intensities of the p-tau bands were dramatically increased in *Akt* cTKO mice. Tau5 antibody was used to detect total tau (t-tau). **b** Immunohistochemistry of p-tau^Thr205^ in the brain. Strong immuno-reactivity of p-tau^Thr205^ was seen in the cortex (e), hippocampal CA3 (f), the hilus of the dentate gyrus (g) and the cerebellum (h) of *Akt* cTKO mice. Weak immuno-reactivity of p-tau^Thr205^ was observed in different brain sub-areas of control mice (a-d)

Increased levels of tau phosphorylated (p-tau) at the Thr205, Thr231 and Ser396 epitopes have been widely reported in Alzheimer’s brain and mouse models [2]. Our Western results showed highly increased levels for p-tau^T205^, p-tau^T231^ and p-tau^S396^ in *Akt* cTKO mice (Fig. 1a). Moreover, the position of p-tau bands at the 60, 64 and 68 kDa in the SDS gels was consistently higher in *Akt* cTKOs than that in controls (Fig. 1a), likely due to the slow migrating rate caused by tau hyperphosphorylation. We conducted additional control experiments and used two groups of *Akt2/3* double KO mice, *Akt1*^*f/f*^*;Akt2*^*−/−*^*;Akt3*^*−/−*^*;CAG-CreER* and *Akt1*^*f/f*^*;Akt2*^*−/−*^*;Akt3*^*−/−*^, which were age- matched littermates to *Akt* cTKOs. We found that *Akt* DKO mice did not show significant changes on p-tau levels (Additional file 1: Figure S2). Strong p-tau^Thr205^ immuno-reactivity was clearly seen in the cytoplasm of neurons in various brain sub-areas of *Akt* cTKOs but not controls (Fig. 1b). Increased p-tau^Ser396^ immuno-reactivity was largely detected in glial-like cells in the brain of *Akt* cTKOs (Additional file 1: Figure S3). Immunostaining on p-tau^Thr231^ showed strong signals in neuronal branches of the cTKO mice (Additional file 1: Figure S4), and double-staining with MAP2 suggested dendritic localization for p-tau^Thr231^ (Additional file 1: Figure S4C: a-d).

TUNEL assay was performed to examine cell death. No difference in the total number of TUNEL+ cells was observed in brains of *Akt* cTKO and control mice (Additional file 1: Figure S5), suggesting no significant change in apoptosis. Markers for neurons, astrocytes and microglia were used for biochemical and morphological analyses. First, there was no significant reduction on cortical levels for NeuN, GFAP and Iba1 in *Akt* cTKO mice (Additional file 1: Figure S6A). Second, there was comparable immuno-reactivity for NeuN, GFAP or Iba1 (Additional file 1: Figure S6B-C) in control and *Akt* cTKO mice. Therefore, deletion of Akt does not significantly affect the survival of cortical cells.

**Fig. 2:**
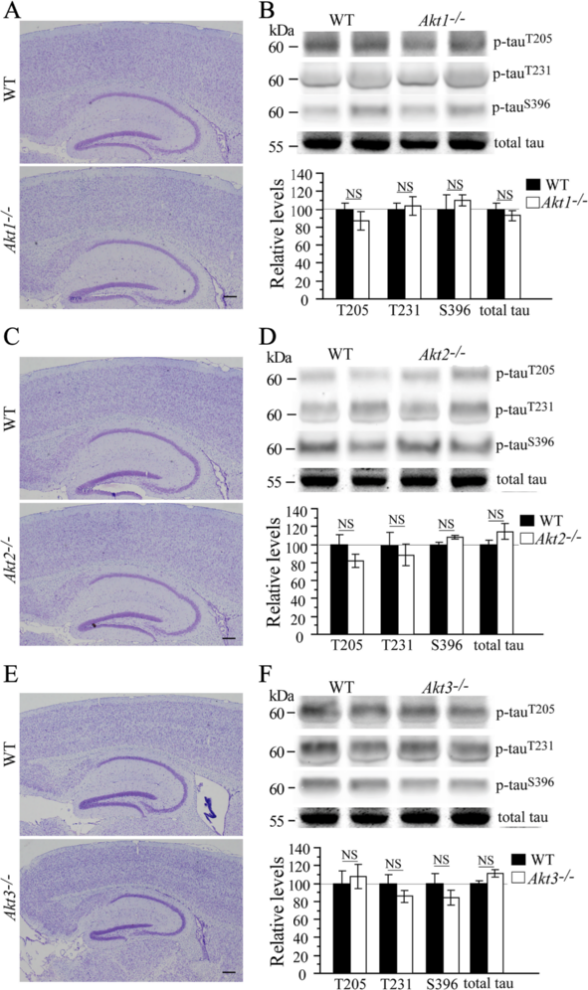
Unchanged levels of p-tau in Akt single isoform KO mice. **a** Nissl staining for the brain of *Akt1* ^−/−^ mice. Normal brain structure was observed. **b** Western analyses on p-tau levels using cortical samples of *Akt1* ^−/−^ mice. There was no significant difference on relative levels of p-tau^Thr205^, p-tau^Thr231^ and p-tau^Ser396^ in *Akt1* ^−/−^ mice. **c** Nissl staining for the brain of *Akt2* ^−/−^ mice. No abnormal brain structure was detected. **d** Western analyses on p-tau levels using cortical samples of *Akt2* ^−/−^ mice revealed no significant difference. **e** Nissl staining for the brain of *Akt3* ^−/−^ mice. There was a smaller brain in *Akt3* ^−/−^ mice than in WT animals. **f** Western analyses on p-tau levels showed no significant difference in *Akt3* ^−/−^ mice (NS = not significant)

To determine the role of Akt single isoform in tau phosphorylation in the brain, we analyzed p-tau levels using cortical lysates from three lines of Akt single isoform KO mice including *Akt1*^*−/−*^ [3], *Akt2*^*−/−*^ [4] and *Akt3*^*−/−*^ [5]. Nissl staining revealed comparable brain structure between *Akt1*^*−/−*^, *Akt2*^*−/−*^, *Akt3*^*−/−*^ and their age-matched WT littermates (Fig. 2). Consistent with previously published observations [5, 6], *Akt3*^*−/−*^ mice displayed smaller brain than WT (Fig. 2e). However, no significant changes in p-tau levels were observed in *Akt1*^*−/−*^, *Akt2*^*−/−*^ and *Akt3*^*−/−*^ mice (Fig. 2b,d,f: ps > 0.05), suggesting that tau hyperphosphorylation shown in *Akt* cTKO mice is unlikely to be caused by loss of a single Akt isoform.

**Fig. 3:**
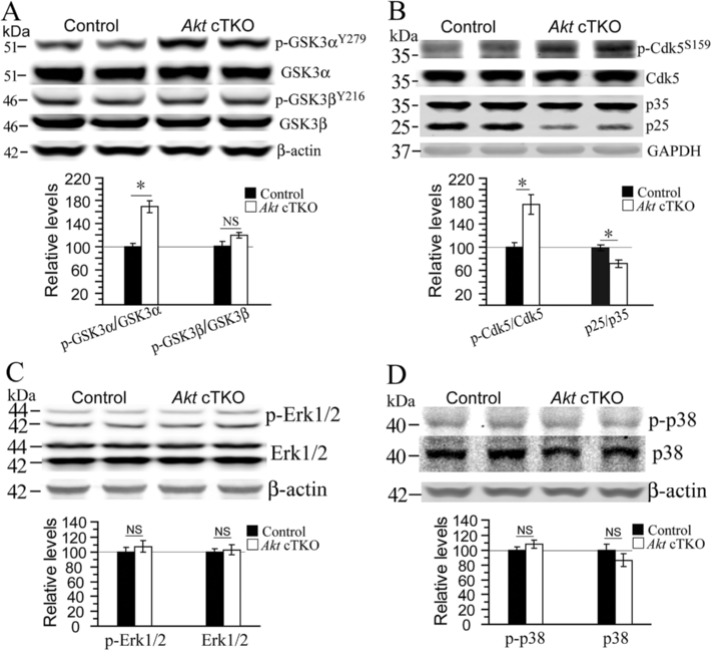
Increased levels for an activated form of GSK3α in the brain of *Akt* cTKO mice. **a** Western blotting on activated forms of GSK3α and GSK3β. Quantitative analysis showed significantly increased levels for p-GSK3α^Tyr279^ (*, p < 0.05) but unchanged levels for p-GSK3β^Tyr216^ (NS, p > 0.05) in *Akt* cTKO mice. **b** Western blotting on p-Cdk5, p35 and p25. Relative levels for p-Cdk5^Ser159^ were significantly increased (*, p < 0.05), but those for p25 were significantly decreased (*, p < 0.05) in the cortex of *Akt* cTKO mice. GAPDH served as the loading control. **c** Western blotting on MAPK Erk1/2. Total Erk1/2 levels in *Akt* cTKO mice were not different from those in control mice. β-actin served as the loading control. Ratio of p-Erk1/2 to the total Erk1/2 in *Akt* cTKO mice was also not different from that in control mice (p > 0.05). **d** Western blotting on phosphorylated MAPK p38 (p-p38). Levels of total p38 did not differ between *Akt* cTKO and control mice (p > 0.05). Ratio of p-p38 to total p38 in *Akt* cTKO mice was not changed (NS = not significant)

To dissect molecular pathways involved, we conducted Western blotting using an antibody against phosphorylated Akt substrates. We found increased intensities for several bands including those for GSK3α/3β in *Akt* cTKO mice (Additional file 1: Figure S7A). Since GSK3 is a substrate of Akt, it is expected that deletion of Akt may cause reduced levels for phosphorylated GSK3α/3β [7]. However, we found that relative levels for p-GSK3β^Ser9^ were significantly increased in *Akt* cTKO mice (Additional file 1: Figure S7B) and those for p-GSK3α^Tyr279^ were also increased (Fig. 3a), suggesting inhibition of GSK3β but activation of GSK3α. Since β-catenin is a well-known substrate of GSK3 [8, 9], p-β-catenin^Ser33/Ser37/Thr41^ was examined and showed significantly increased levels in *Akt* cTKOs (Additional file 1: Figure S7C), suggesting elevated GSK3α activity. Moreover, we examined several other tau kinases. First, although levels for p-Cdk5^Ser159^ were increased, those for p25 were decreased (Fig. 3b, p < 0.05). Second, no significant changes on levels for p-Erk1/2 and p-p38 were detected in *Akt* cTKO mice (Fig. 3c-d, ps > 0.05). Overall, activities for GSK3β, Cdk5, Erk and MAPK p38 were not enhanced.

**Fig. 4:**
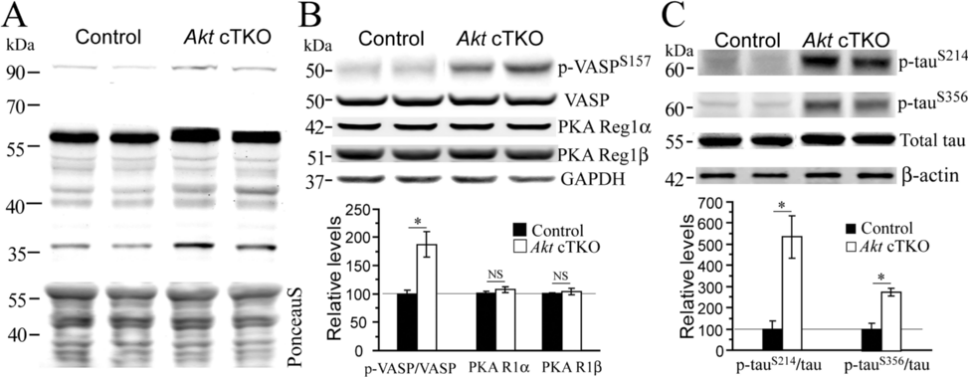
Increased levels for phosphorylated PKA substrates in *Akt* cTKO mice. **a** Western blotting for phosphorylated PKA substrates using cortical samples of *Akt* cTKO mice. Levels for several phosphorylated PKA substrates with molecular weights at about 100 kDa, 60 kDa and 40 kDa were increased. **b** Western analyses on VASP and PKA subunits. P-VASP^**Ser157**^ levels in *Akt* cTKO mice were highly increased but total VASP levels were not changed. Relative levels for PKA regulatory subunits, 1α and 1β, were not changed. GAPDH served as the loading control. **c** Western blotting on p-tau^Ser214^ and p-tau^Ser356^. Ratios of p-tau to total tau were significantly increased in *Akt* cTKO mice, as compared to control animals (*, p < 0.05)

It has been shown that GSK3β is phosphorylated at the Ser9 site not only by Akt but also by PKA [10]. The tyrosine phosphorylation of GSK3 requires the cAMP-PKA signaling [11, 12]. Moreover, it is known that PKA is a kinase to phosphorylate tau at the sites of Thr205, Thr231 and Ser396 [2, 13]. To test the possibility that PKA is involved, we performed the following experiments. First, an antibody against phosphorylated PKA substrates was used to conduct Western blotting. Increased levels for several bands with a wide range of molecular weights were observed (Fig. 4a), suggesting increased PKA activity. Second, we examined VASP, a well-known PKA substrate [14, 15], and observed highly increased p-VASP^Ser157^ levels (Fig. 4b). In contrast, expression levels of PKA regulatory subunits, 1α and 1β, were unchanged (Fig. 4b). Third, since the Ser214 [16] and Ser356 [17, 18] sites of tau are phosphorylated by PKA as well, we analyzed p-tau^Ser214^ and p-tau^Ser356^, which exhibited increased levels in *Akt* cTKO mice (Fig. 4c).

## Discussion

Akt levels are decreased in Alzheimer’s brain [19]. However, it remains unknown exactly how Akt is involved in pathophysiological processes of AD, e.g. tau pathology and neurodegeneration. In this study, a novel *Akt* cTKO mouse model was generated and displayed tau hyperphosphorylation in the brain. We demonstrated that *Akt* cTKO mice exhibited significantly increased levels for several PKA downstream substrates. We observed increased levels for an activated form of GSK3α in *Akt* cTKO mice. We reported unchanged levels for activated forms of GSK3β, Cdk5, Erk and MAPK p38 in *Akt* cTKO mice.

It had been difficult to study normal physiological functions of Akt in the adult brain. First, while there are three Akt isoforms in mammals [20], a recent study did not identify which Akt isoform plays important role in neurodegenerative disease [19]. Second, since global deletion of Akt three isoforms causes early embryonic death in mice [1], a viable *Akt* cTKO mouse model was generated to overcome developmental problems in this study. Interestingly, *Akt* cTKO animals but not Akt single isoform KO mice exhibited increased p-tau levels in the brain, suggesting that total Akt but not a single isoform plays a key role in tau phosphorylation. Immunostaining using AT8 or AT100 antibodies revealed no NFTs-like structure in *Akt* cTKO mice (data not shown). No significant changes on the total number of TUNEL+ cells or NeuN+ cells were detected in *Akt* cTKO mice. These findings are consistent with the concept that tau hyperphosphorylation is an early event during neurodegenerative process [21].

Tau phosphorylation status is determined by activities of multiple protein kinases [22]. Since there were unchanged levels for activated forms of GSK3β, Cdk5, Erk and p38, it is unlikely that they could account for abnormal tau phosphorylation in *Akt* cTKO mice. Although it is believed that inactivation of Akt may cause reduced p-GSK3α^S21^/3β^S9^ [7] and thus lifts the inhibition on GSK3, levels for p-GSK3β^S9^ were actually increased in *Akt* cTKO mice. Since GSK3β is a substrate for both Akt and PKA [10, 12], in the absence of Akt, it can be phosphorylated by PKA. The findings on phosphorylated PKA substrates, p-VASP and p-tau (Fig. 4) suggest enhanced PKA activity, which helps explain why there was tau hyperphosphorylation in *Akt* cTKO mice. There are two possible mechanisms by which Akt regulates PKA activity. The first possibility is a compensatory mechanism. It is known that the PI3K-Akt [23] and PKA pathways [24] can be activated by extracellular signal molecules such as cytokines. In the absence of the PI3K-Akt pathway, such molecules may over-activate the cAMP-PKA signaling as compensation. The second mechanism is that phosphodiesterases (PDEs) might be involved in the regulation of PAK activity. PDEs are Akt substrates [25] and can degrade cAMP, the second messenger molecule [26]. Therefore, deletion of Akt may inhibit PDEs and cAMP levels may get increased. Upon activation by cAMP, PKA can act on various downstream targets including tau, GSK3 and VASP.

## Methods

### Generation of *Akt1*^*f/f*^ and *Akt* cTKO mice

We used a strategy shown in Additional file 1: Figure S1A to generate floxed *Akt1* mice. The detailed breeding plan for the generation of *Akt* cTKO mice was described in the Additional file 1.

### Treatment of mice with tamoxifen

Tamoxifen was used to treat *Akt1*^*f/f*^*;Akt2*^*−/−*^*;Akt3*^*−/−*^*;CAG-CreER* and age-matched littermate controls for 5 consecutive days. Detailed information was described in the Additional file 1.

### Brain lysates preparation

Procedures were described previously [27]. Immunoblotting methods and antibody information were described in the Additional file 1.

### Statistical analysis

Data were presented as the mean ± SEM. Two-tailed Student’s *t*-test was performed to examine the difference between control and cKO mice. P < 0.05 (*) was considered statistically significant.

### Ethical approval

Mouse breeding was conducted under IACUC approved protocols at the MARC (Model Animal Research Center). All the experiments were performed in accordance with the Guide for the Care and Use of Laboratory Animals of the MARC at Nanjing University.

## Additional file

Additional file 1:
**Conditional inactivation of Akt three isoforms causes tau hyperphosphorylation in the brain.** (PDF 3519 kb)

## Abbreviations

AD: Alzheimer’s disease
Akt: Protein kinase B
PKA: Protein kinase A
cAMP: Cyclic adenosine 3′5′ monophosphate
*Akt* cTKO: *Akt* three isoforms conditional knockout
PI3K: Phosphatidylinositol-3 kinase
PDE: Phosphodiesterase
tau: Microtubule associated protein
p-tau: Phosphorylated tau
GSK3: Glycogen synthase kinase-3
Cdk5: Cyclin-dependent kinase 5
MAPK: Mitogen-activated protein kinase
Erk: Extracellular signal-regulated kinase
TUNEL: Terminal deoxynucleotidyl transferase-mediated dUTP-biotin nick end labeling
NFTs: Neurofibrillary tangles
MAP2: Microtubule-associated protein
GFAP: Glial fibrillary acidic protein
Iba1: Ionized calcium-binding adapter molecule 1
VASP: Vasodilator stimulated phosphoprotein
