# Supplementary material for: Conditional inactivation of Akt three isoforms causes tau hyperphosphorylation in the brain
Source: Mol Neurodegener. 2015 Jul 31;10:33. doi: 10.1186/s13024-015-0030-y (PMC4521471; doi:10.1186/s13024-015-0030-y)
Supplement: Additional file 1: — Conditional inactivation of Akt three isoforms causes tau hyperphosphorylation in the brain. (PDF 3519 kb) [file 13024_2015_30_MOESM1_ESM.pdf]

Additional file

Conditional inactivation of Akt three isoforms causes tau hyperphosphorylation in the brain

Long Wang[1,¶], Shanshan Cheng[1,¶], Zhenyu Yin[2], Congyu Xu[1], Shuangshuang Lu[1], Jinxing Hou[1], Tingting Yu[2], Xiaolei Zhu[4], Xiaoyan Zou[1], Ying Peng[3], Yun Xu[4,*], Zhongzhou Yang[1,*] and Guiquan Chen[1,*]

1. Model Animal Research Center，MOE Key Laboratory of Model Animal for Disease Study, Nanjing University, 12 Xuefu Avenue, Nanjing, Jiangsu Province, China, 210061.

2. Department of Geriatric, Nanjing Drum Tower Hospital, Nanjing University Medical School, 321 Zhongshan Avenue, Nanjing, Jiangsu Province, China, 210008.

3. Institute of Materia Medica, Chinese Academy of Medical Sciences & Peking Union Medical College, Xuanwu District, Beijing, China, 100050.

4. Department of Neurology, Nanjing Drum Tower Hospital, Nanjing University Medical School, 321 Zhongshan Avenue, Nanjing, Jiangsu Province, China, 210008.

[¶]These authors contribute equally to this work.

[*]**Corresponding authors:** Dr. Guiquan Chen, email: chenguiquan@nju.edu.cn; Prof. Xun Yu, email: xuyun20042001@aliyun.com and Prof. Zhongzhou Yang, email: yangzz@nicemice.cn

Methods

Generation of *Akt1*$^{f/f}$ and *Akt* cTKO mice.

Since *Akt1*$^{-/-}$ mice are perinatal lethal [1, 2], we generated floxed *Akt1* mice. The gene targeting strategy was shown in Fig. S1A. Two loxP sites were inserted into introns 2 and 11 separately. After obtaining *Akt1*$^{f/+}$, we intercrossed them to get *Akt1*$^{f/f}$. To generate viable *Akt* cTKO mice, first, we crossed *Akt2*$^{-/-}$ [3] with *Akt3*$^{-/-}$ [4] to obtain *Akt2*$^{+/-}$;*Akt3*$^{+/-}$. The latter were bred with *Akt1*$^{f/f}$ to get *Akt1*$^{f/+}$;*Akt2*$^{+/-}$;*Akt3*$^{+/-}$, which were bred with a *CAG-CreER* to get *Akt1*$^{f/+}$;*Akt2*$^{+/-}$;*Akt3*$^{+/-}$;*CAG-CreER*. All the mice were bred in an SPF room in the core animal facility of the Model Animal Research Center (MARC) at Nanjing University. The room temperature was 25ºC and the light-cycle was automatically controlled. The genetic background of the mice used in this study is C57BL/6. The protocol for the study has been approved by the institutional Animal Care and Use Committee of the MARC.

We crossed *Akt1*$^{f/+}$;*Akt2*$^{+/-}$;*Akt3*$^{+/-}$;*CAG-CreER* with *Akt1*$^{f/+}$;*Akt2*$^{+/-}$;*Akt3*$^{+/-}$ or *Akt1*$^{f/f}$;*Akt2*$^{+/-}$;*Akt3*$^{+/-}$ to get *Akt1*$^{f/f}$;*Akt2*$^{-/-}$;*Akt3*$^{-/-}$;*CAG-CreER* and littermates with a number of genotypes. Since there were quite many gene combinations (54) in the offspring's genotypes, it was unlikely to use all of them as the control for *Akt* cTKO. We kept age-matched littermates with the following genotypes: *Akt1*$^{f/f}$;*Akt2*$^{+/+}$;*Akt3*$^{+/+}$, *Akt1*$^{f/+}$;*Akt2*$^{+/+}$;*Akt3*$^{+/+}$, *Akt1*$^{f/f}$;*Akt2*$^{-/-}$;*Akt3*$^{-/-}$ and *Akt1*$^{f/f}$;*Akt2*$^{-/-}$;*Akt3*$^{-/-}$;*CAG-CreER* (no tamoxifen treatment) as the control. In Figure 1, we run p-tau Western on *Akt* cTKO vs *Akt1*$^{f/f}$;*Akt2*$^{+/+}$;*Akt3*$^{+/+}$ and *Akt1*$^{f/+}$;*Akt2*$^{+/+}$;*Akt3*$^{+/+}$. In Figure S2, Western blotting

on p-tau for *Akt2/3* DKO (*Akt1$^{f/f}$;Akt2$^{-/-}$;Akt3$^{-/-}$;CAG-CreER* and *Akt1$^{f/f}$;Akt2$^{-/-}$;Akt3$^{-/-}$*) mice was presented.

Treatment of mice with tamoxifen.

To induce Cre-mediated gene recombination, *Akt1$^{f/f}$;Akt2$^{-/-}$;Akt3$^{-/-}$;CAG-CreER* and controls were treated with tamoxifen for 5 consecutive days. Tamoxifen was purchased from Sigma-Aldrich (catalogue No. T5648), and was freshly prepared in each injection day. The treatment was conducted by intraperitoneal (i.p.) injection of the drug at the concentration of 20mg/kg. *Akt1$^{f/f}$;Akt2$^{-/-}$;Akt3$^{-/-}$;CAG-CreER* mice receiving 5 doses of tamoxifen injections were designated as *Akt* cTKO mice. The number of animals used for the *Akt* cTKO study was as follows: control=7, *Akt* cTKO=9. For Akt single isoform KO studies, the number was 3-4 per genotypic group.

Genotyping.

To detect the floxed *Akt1* allele, the following primers were used: GGGATCAGCAG-TTGAAGGACAGA and GCCAGGAATACAGCATGAGCCAC. The PCR products for the WT and the floxed allele are 196bp and 302 bp, respectively. To detect *Akt1*$^{\Delta/\Delta}$ allele, primers of AGACTCTGAGCATCATCCCTGGG, CGTCTGGCCTTCCTGTA-GCCAG and TGAAGCAGGCCTAGAGCCCCATG were used. The PCR product for the WT allele is 300bp and that for the $^{\Delta}$ allele is 200 bp. To identify *Akt2*$^{\Delta/\Delta}$ allele, primers of CTCAGGGACACCCATGTGTGGCTGC, GCTGCCTCGTCCTGCAGT-

TCATTC and CCACAGGCAGCAGAAAGGAA were used. The PCR size for the WT allele is 360bp and that for the $^{\Delta}$ allele is 600 bp. For *Akt3*$^{\Delta/\Delta}$ mice, primers used were: GGTTCTGTGGGAGGTAGTTCTC, GCAATCCATCTTGTTCAATGGCCG and CCATCGGTCGGCTACGGCTTGG. The PCR band for the WT allele is 500bp and that for the $^{\Delta}$allele is 350 bp.

Nissl staining.

Sagittal brain sections (10μm) were de-paraffinized, ethanol dehydrated, and were then washed using distilled water. Sections were treated with 0.5% cresyl-violet for 10 minutes and then washed with PBS. Sections were incubated in a solution containing 1% glacial acetic acid and 16% ethanol. Sections were dehydrated using an ascending series of ethanol (70%, 90%, 95% and 100%), and were then placed in toluene. Slides were coverslipped using neutral resin.

Brain lysates preparation.

The mice for experiments were euthanized by CO_2. For each *Akt* isoform KO line, mice were sacrificed at 2-3 months of age. For the *Akt* cTKO study, control and mutant mice were sacrificed 3 weeks after the tamoxifen treatment. After euthanasia, different brain areas such as the cortex and the cerebellum were quickly dissected out and were immediately put into liquid nitrogen. Brain samples were stored at -80℃ until use. Mice cortices were homogenized in cold radio immunoprecipitation assay lysis buffer [consisting of the following (in mM): 20mM Tris-HCl, pH 7.4, 150mM

NaCl, 1mM EDTA, 1% NP-40, 0.5% sodium deoxycholate, and 0.1% SDS] containing protease and phosphatase inhibitors (Thermo). Lysates were cleared by centrifugation (14,000 rpm for 20 min). Protein concentration was analyzed using a standard BSA method [5, 6].

Immunoblotting.

Normalized volumes of samples (30μg total protein) were resolved in 10% SDS-PAGE (invitrogen), transferred to nitrocellulose membrane. After blocking with 5% (w/v) dry milk for 1h, membranes were incubated with primary antibodies overnight and detected using infrared dye-coupled secondary antibodies (goat anti-rabbit IRdye800, goat anti-rabbit IRdye680, goat anti-mouse IRdye800 and goat anti-mouse IRdye680). Membranes were scanned and data were quantified using Odyssey Infrared Imaging System (Li-Cor). Antibody against total Akt (t-Akt) (1:1000) was made by the Z.Y. lab in the MARC. We purchased the following antibodies from the CST: anti-pAkt^{473} (1:600), anti-GSK3α/β (1:1000), anti-pGSK3α^{Y279}/β^{Y216} (1:600), anti-pGSK3α^{S21} (1:600), anti-pGSK3β^{S9} (1:600), anti-phosphorylated Akt substrates (1:500), anti-pErk1/2 (1:1000), anti-Erk1/2 (1:1000), anti-p-p38 (1:500), anti-p38 (1:1000), anti-MEK1/2 (1:1000), anti-pMEK1/2 (1:1000), anti-phosphorylated PKA substrates (1:500), anti-p-β-catenin$^{Ser33/Ser37/Thr41}$ (1:600), anti-β-catenin (1:1000), anti-c-Raf (1:1000) and anti-p-c-Raf (1:1000). Other antibodies were listed as follows: anti-pCdk5 (1:100, Santa Cruz); anti-Cdk5 (1:200, Santa Cruz), anti-PKA reg 1α&1β (1:1000, Santa Cruz), anti-pVASP^{Ser157} (1:600, Santa Cruz), anti-p35/p25 (1:250,

Santa Cruz), anti-VASP (1:1000, Bioworld), anti-ptau^{Thr205} (1:200, Invitrogen), anti-ptau^{Thr231} (1:200, Millipore), anti-ptau^{Ser396} (1:200, Invitrogen), anti-ptau^{Ser214} (1:250, Thermo), anti-ptau^{Ser356} (1:500, Sangon Biotech), HT7 (1:200, Thermo), Tau5 (1:200, Millipore), anti-NeuN (1:500, Millipore), anti-GFAP (1:500, Sigma-Aldrich), anti-Iba1(1:500, Wako), anti-β-actin (1:10000, Sigma-Aldrich) and anti-GAPDH (1:10000, Genetex).

Immunohistochemistry.

Brains were perfused with PBS, fixed in 4% paraformaldehyde, processed for paraffin embedding, and serially sectioned (10μm). Sagittal sections were deparaffinized, ethanol dehydrated, and immunostained with antibodies raised specifically against total Akt (1:200), NeuN (1:500), microtubule-associated protein 2 (MAP2) (1:200, Sigma-Aldrich), GFAP(1:500), Iba1(1:500), ptau^{Thr205} (1:1000), ptau^{Thr231} (1:10000) and ptau^{Ser396} (1:200). For fluorescence immunostaining, brain sections were incubated with either Alexa Fluor 488 goat anti-mouse/anti-rabbit or Alexa Fluor 594 goat anti-mouse/anti-rabbit secondary antibodies (Invitrogen), and then analyzed with a Leica confocal laser-scanning microscope.

TUNEL staining.

The brain sections were blocked using 5% of goat serum for 30 min followed by the treatment of Fluorescein (Roche) at 37 ºC for an hour [7, 8]. The slides were then washed using TBS (tris-buffered saline) for three times. A brain section from

4-months-old neuron-specific *Dicer* conditional mouse was used as a positive control, which displayed many TUNEL positive(+) cells [9]. TUNEL staining was scanned using a Leica confocal laser microscope.

References

1. Yang, ZZ, Tschopp, O, Hemmings-Mieszczak, M, Feng, J, Brodbeck, D, Perentes, E, et al. Protein kinase B alpha/Akt1 regulates placental development and fetal growth. J Biol Chem. 2003;278:32124-31.
2. Cho, H, Thorvaldsen, JL, Chu, Q, Feng, F & Birnbaum, MJ. Akt1/PKBalpha is required for normal growth but dispensable for maintenance of glucose homeostasis in mice. J Biol Chem. 2001;276:38349-52.
3. Cho, H, Mu, J, Kim, JK, Thorvaldsen, JL, Chu, Q, Crenshaw, EB, 3rd, et al. Insulin resistance and a diabetes mellitus-like syndrome in mice lacking the protein kinase Akt2 (PKB beta). Science. 2001;292:1728-31.
4. Tschopp, O, Yang, ZZ, Brodbeck, D, Dummler, BA, Hemmings-Mieszczak, M, Watanabe, T, et al. Essential role of protein kinase B gamma (PKB gamma/Akt3) in postnatal brain development but not in glucose homeostasis. Development. 2005;132:2943-54.
5. Chen, G, Zou, X, Watanabe, H, van Deursen, JM & Shen, J. CREB binding protein is required for both short-term and long-term memory formation. J Neurosci. 2010;30:13066-13077.
6. Saura, CA, Chen, G, Malkani, S, Choi, S-Y, Takahashi, RH, Zhang, D, et al. Conditional inactivation of presenilin 1 prevents amyloid accumulation and temporarily rescues contextual and spatial working memory impairments in amyloid precursor protein transgenic mice. J Neurosci. 2005;25:6755-6764.
7. Zhang, C, Wu, B, Beglopoulos, V, Wines-Samuelson, M, Zhang, D, Dragatsis, I, et

al. Presenilins are essential for regulating neurotransmitter release. Nature. 2009;460:632-6.

8. Tabuchi, K, Chen, G, Sudhof, TC & Shen, J. Conditional forebrain inactivation of nicastrin causes progressive memory impairment and age-related neurodegeneration. J Neurosci. 2009;29:7290-7301.
9. Cheng, S, Zhang, C, Xu, C, Wang, L, Zou, X & Chen, G. Age-dependent neuron loss is associated with impaired adult neurogenesis in forebrain neuron-specific Dicer conditional knockout mice. Int J Biochem Cell. 2014;57:186-196.

Figure S1

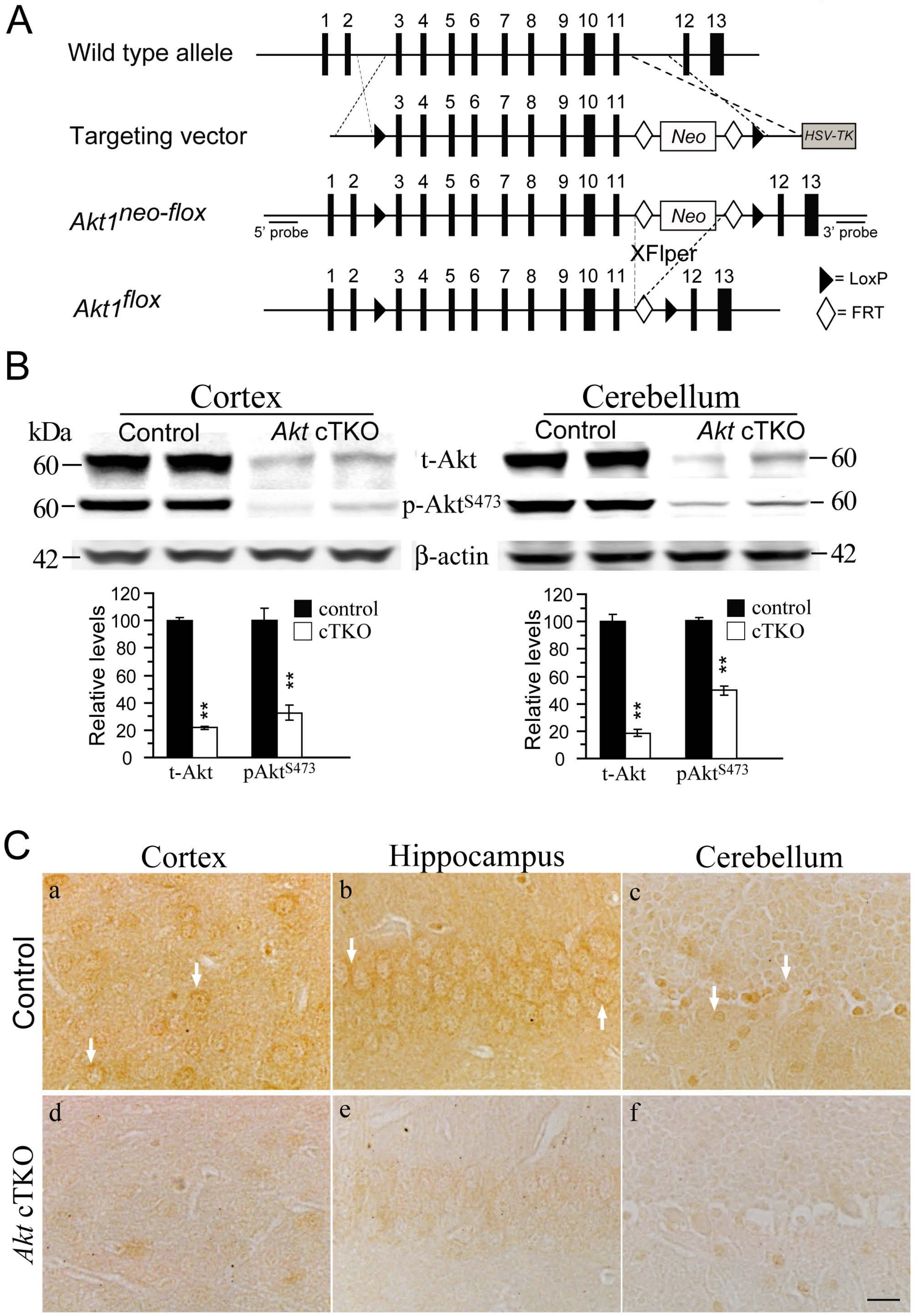
A
Wild type allele
Targeting vector
Neo
HSV-TK
Akt1neo-flox
5' probe
3' probe
XFlper
Akt1flox
= LoxP
= FRT
B
Cortex
Cerebellum
kDa
Control
Akt cTKO
t-Akt
p-AktS473
β-actin
60
42
Relative levels
control
cTKO
t-Akt
pAktS473
C
Cortex
Hippocampus
Cerebellum
Control
Akt cTKO
a
b
c
d
e
f

Supplementary Figure 1. Molecular characterization of tamoxifen-induced *Akt* cTKO mice. (**A**) Gene targeting strategy for the generation of floxed *Akt1* mice. In the targeting vector, a neomycin resistance cassette (NEO), FRT sites (open diamonds) and loxP sites (arrowheads) were indicated. A herpes simplex virus-thimidine kinase (HSV-TK) was used for negative selection. Several crossings were made to obtain $Akt1^{f/f};Akt2^{-/-};Akt3^{-/-};CAG$-*CreER* which were designated as *Akt* cTKO after the treatment of tamoxifen. (**B**) Western blotting on total Akt (t-Akt) and phosphorylated Akt (p-Akt) in the brain of *Akt* cTKO mice. In the cortex, levels of t-Akt were markedly reduced (control=100±2.0%, cTKO=21.8±1.1%, p<0.01, two-tailed Student t-test). In the cerebellum, levels of t-Akt were reduced (control=100±5.4%, cTKO=18.6±2.6%, p<0.01). In the cortex, levels of p-Akt^{Ser473} were decreased (control=100±9.1%, cTKO=32.6±5.7%, p<0.01). In the cerebellum, levels of p-Akt^{Ser473} were decreased (control=100±2.5%, cTKO= 49.5± 3.4%, p<0.01). The control mice were $Akt1^{f/f};Akt2^{+/+};Akt3^{+/+}$ and $Akt1^{f/+};Akt2^{+/+};Akt3^{+/+}$. β-actin served as the internal control. (**C**)Immunohistochemistry of t-Akt using brain sections of *Akt* cTKO mice. Strong positive immuno-reactivity of the t-Akt was observed in pyramidal neurons in the cortex (a) and the hippocampus (b), and granule cells in the cerebellum (c) of control mice. Faint immuno-reactivity of t-Akt was seen in neurons of the cortex (d), the hippocampus (e) and the cerebellum (f) of *Akt* cTKO mice. **, p<0.01. Scale bar=20μm.

Figure S2

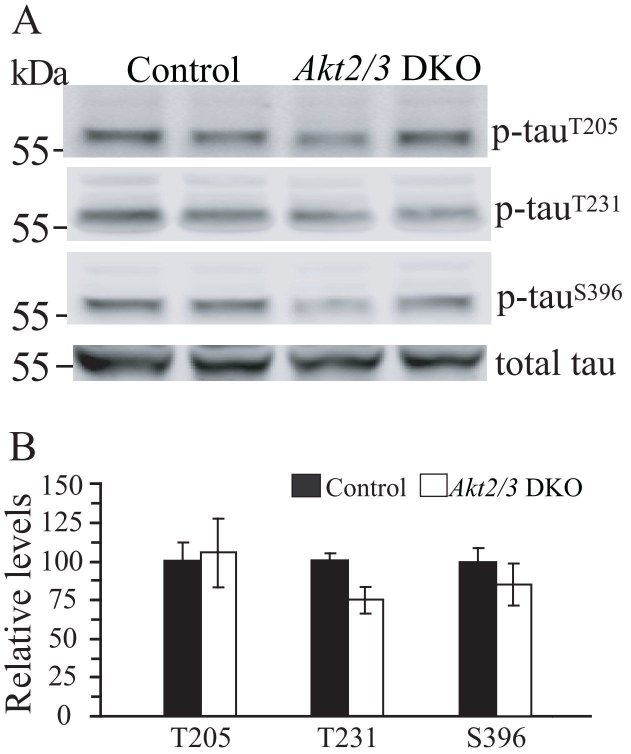

Supplementary Figure 2. **Unchanged p-tau levels in *Akt2/3* double knockout (DKO) mice.** (**A**)Western blotting on p-tau using cortical samples of $Akt1^{f/f};Akt2^{-/-};Akt3^{-/-}$ and $Akt1^{f/f};Akt2^{-/-};Akt3^{-/-};CAG\text{-}CreER$ (without tamoxifen treatment). These two genotypes were age-matched littermates to *Akt* cTKO, and were designated as *Akt2/3* DKO. The control here referred to $Akt1^{f/f};Akt2^{+/+};Akt3^{+/+}$ and $Akt1^{f/+};Akt2^{+/+};Akt3^{+/+}$.

(**B**)Quantification of p-tau relative to total tau. There were no significant differences on levels for p-tau^{Thr205} (control=100±12.6%, DKO=105.6±22.2%, p>0.5), p-tau^{Thr231} (control=100±4.8%, DKO=74.4±8.4%, p=0.06) and p-tau^{Ser396} (control=100±8.5%, DKO=84.9±24.2%, p>0.5) in the cortex of control and *Akt2/3* DKO mice.

Figure S3

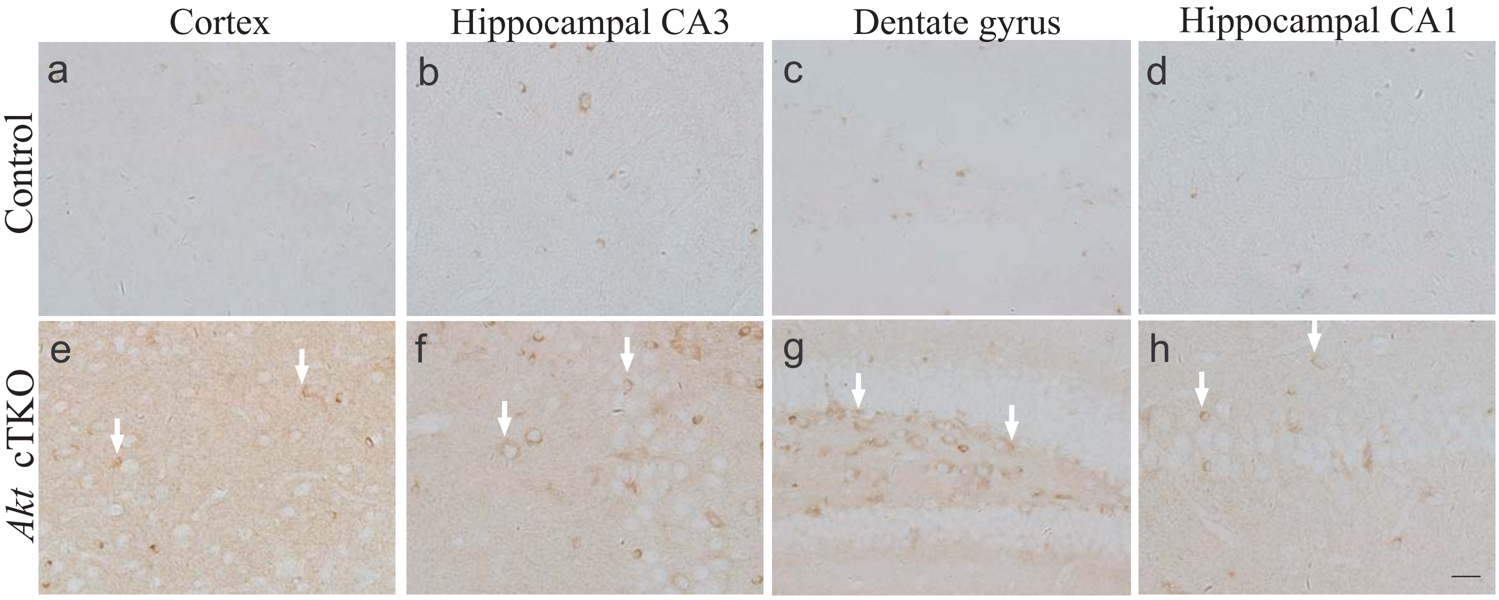

Supplementary Figure 3. Immunohistochemistry of p-tau in the brain of *Akt* cTKO mice. Increased immuno-reactivity of p-tau^{Ser396} was found in the cortex (e), hippocampal CA3 (f), the dentate gyrus (g) and hippocampal CA1 (h) of *Akt* cTKO mice. Scale bar=20μm.

Figure S4

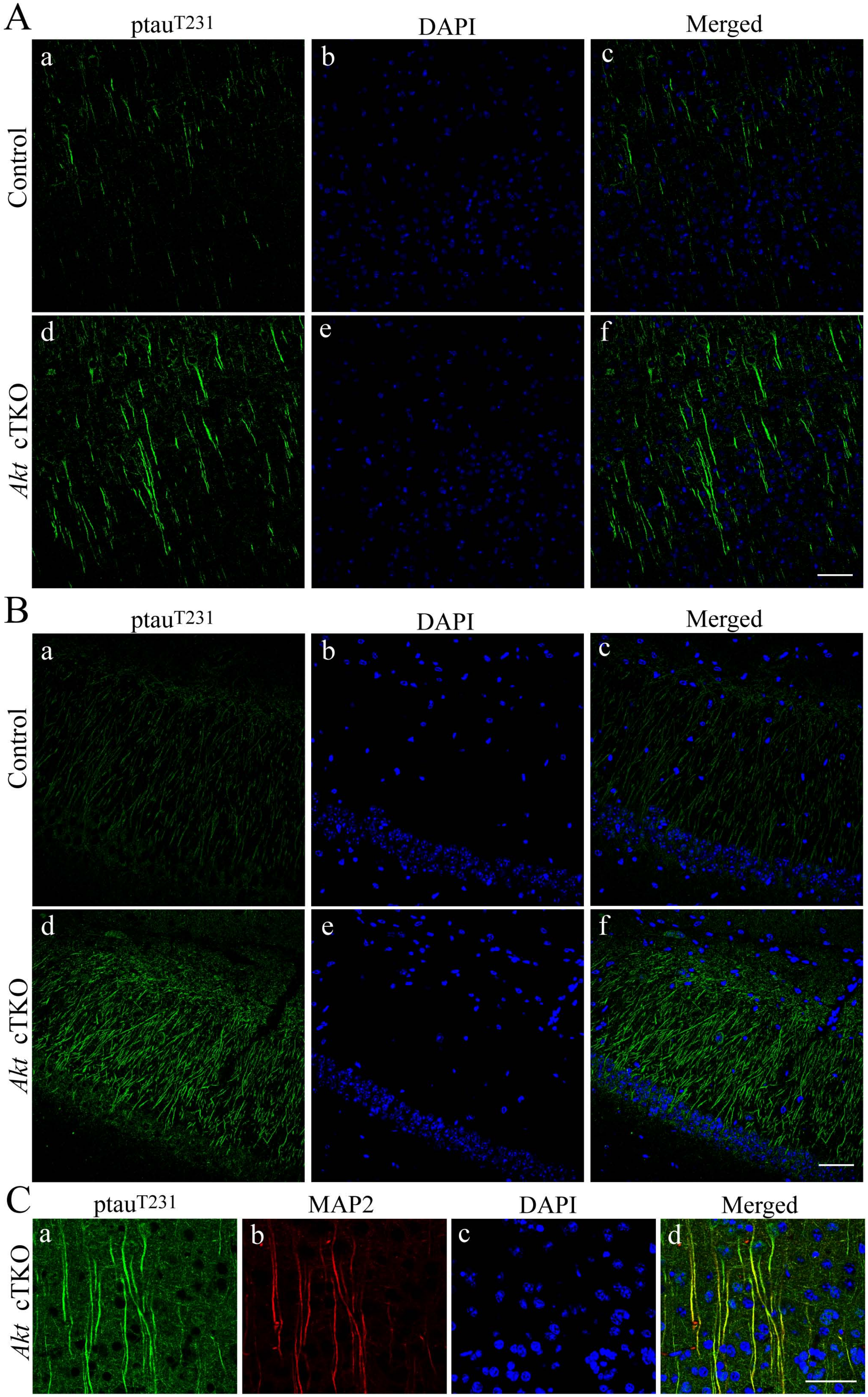
A
ptauT231
DAPI
Merged
a
b
c
Control
d
e
f
Akt cTKO
B
ptauT231
DAPI
Merged
a
b
c
Control
d
e
f
Akt cTKO
C
ptauT231
MAP2
DAPI
Merged
a
b
c
d
Akt cTKO

Supplementary Figure 4. Tau hyperphosphorylation in the brain of *Akt* cTKO mice. (**A**) Immunostaining of p-tau^{Thr231} in the cortex. Strong immunoreactivity of p-tau^{Thr231} was observed in *Akt* cTKO mice (d) as compared to control mice (a). DAPI staining showed comparable number of DAPI+ cells in the cortex of control (b) and *Akt* cTKO (e) mice. (**B**)Immunostaining of p-tau^{Thr231} in the hippocampus. Strong immuno-reactivity of p-tau^{Thr231} was seen in *Akt* cTKO mice (d). There was comparable number of DAPI+ cells in hippocampal CA1 area of control (b) and *Akt* cTKO (e) mice. (**C**)Double-immunostaining of p-tau^{Thr231} and MAP2. P-tau^{Thr231} signals (a) were largely overlapped with those of MAP2 (b). DAPI was shown in (c), and the merged image was shown in (d). Scale bar=50μm.

Figure S5

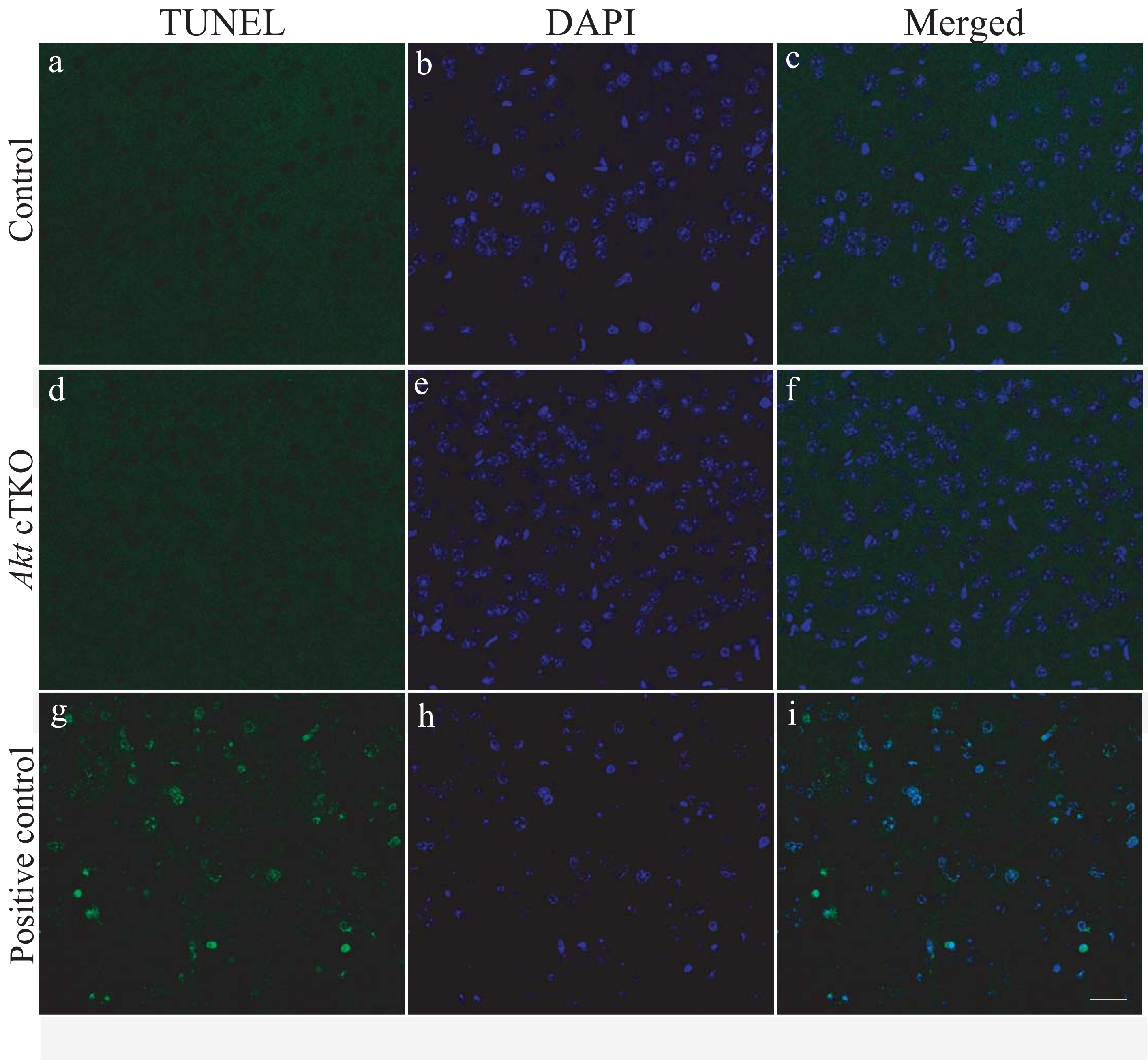

Supplementary Figure 5. **No increased apoptotic cell death in *Akt* cTKO mice.** TUNEL staining on brain sections of *Akt* cTKO mice. There was no difference on the total number of TUNEL positive (+) cells between control (a: TUNEL; b: DAPI; c: merged) and *Akt* cTKO (d: TUNEL; e: DAPI; f: merged) mice. DAPI staining showed comparable number of DAPI+ cells in the cortex of control and *Akt* cTKO mice. (g-i) Brain sections of a forebrain neuron-specific *Dicer* cKO mouse were used as the positive control. Abundant TUNEL+ (g) cells were observed in the cortex of a 4-month-old *Dicer* cKO mouse. DAPI+ (h-i) cells were seen in the same brain section of the *Dicer* cKO mouse. Scale bar=25µm.

Figure S6

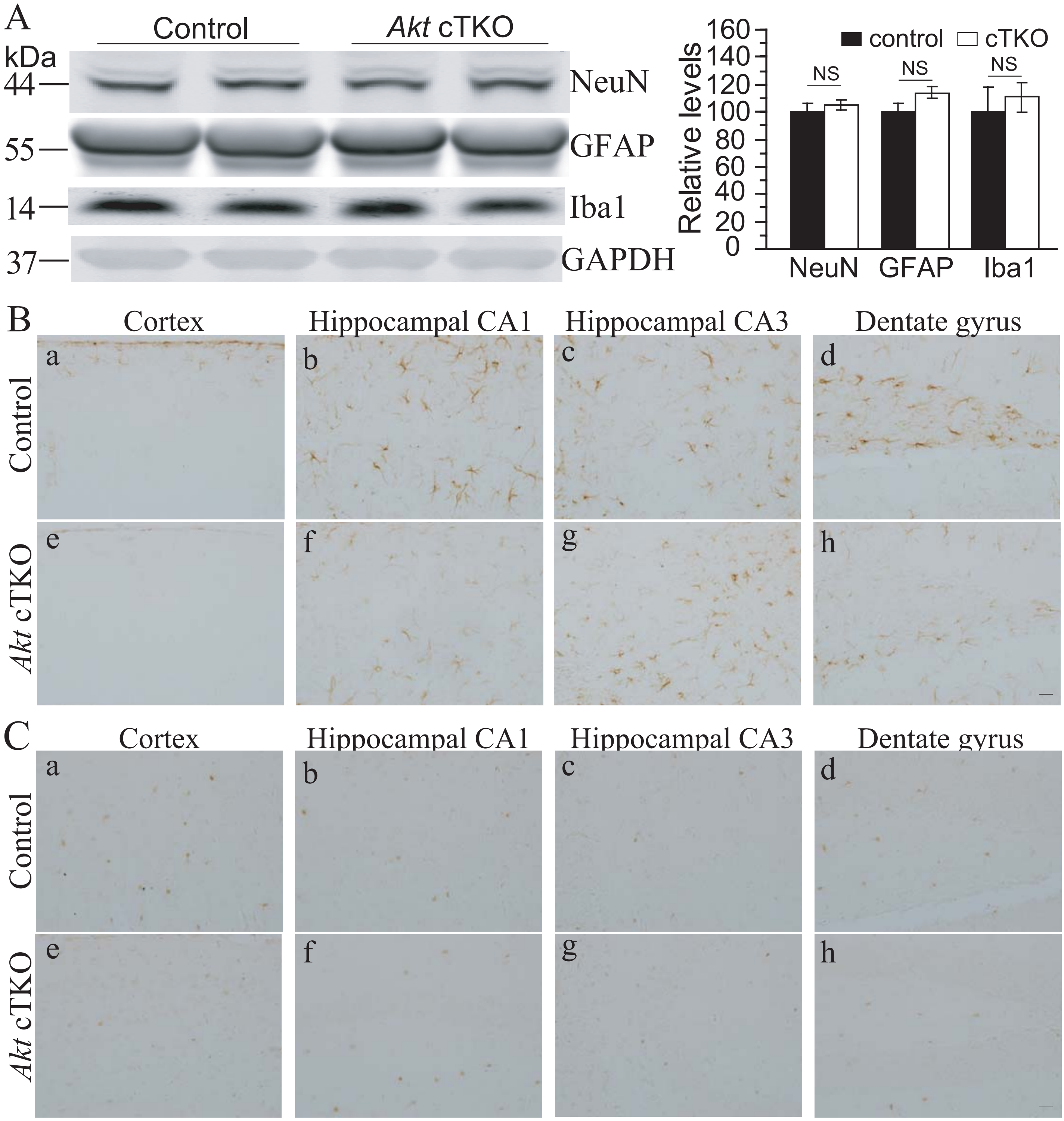

Supplementary Figure 6. No significant cell loss in the brain of *Akt* cTKO mice.

(**A**)Biochemical analyses on markers for different cell types using cortical homogenates. There were no differences on protein levels for NeuN (control=100±6.4%, cTKO=105.1 ±3.6%, p>0.5), GFAP (control=100±6.1%, cTKO=114.2±4.7%, p>0.1) and Iba1(control =100±23.6, cTKO=110.8±13.9%, p>0.5) in the cortex (NS=not significant).

(**B**)Immunohistochemistry of GFAP. There was no detectable astrocytosis in the cortex (e), hippocampal CA1(f), hippocampal CA3 (g) and the dentate gyrus (h) of *Akt* cTKO mice, as compared to the control (a-d). (**C**)Immunohistochemistry of Iba1. There was no significant activation on microglia in the brain (e-h) of *Akt* cTKO mice. Scale bar=20µm.

Figure S7

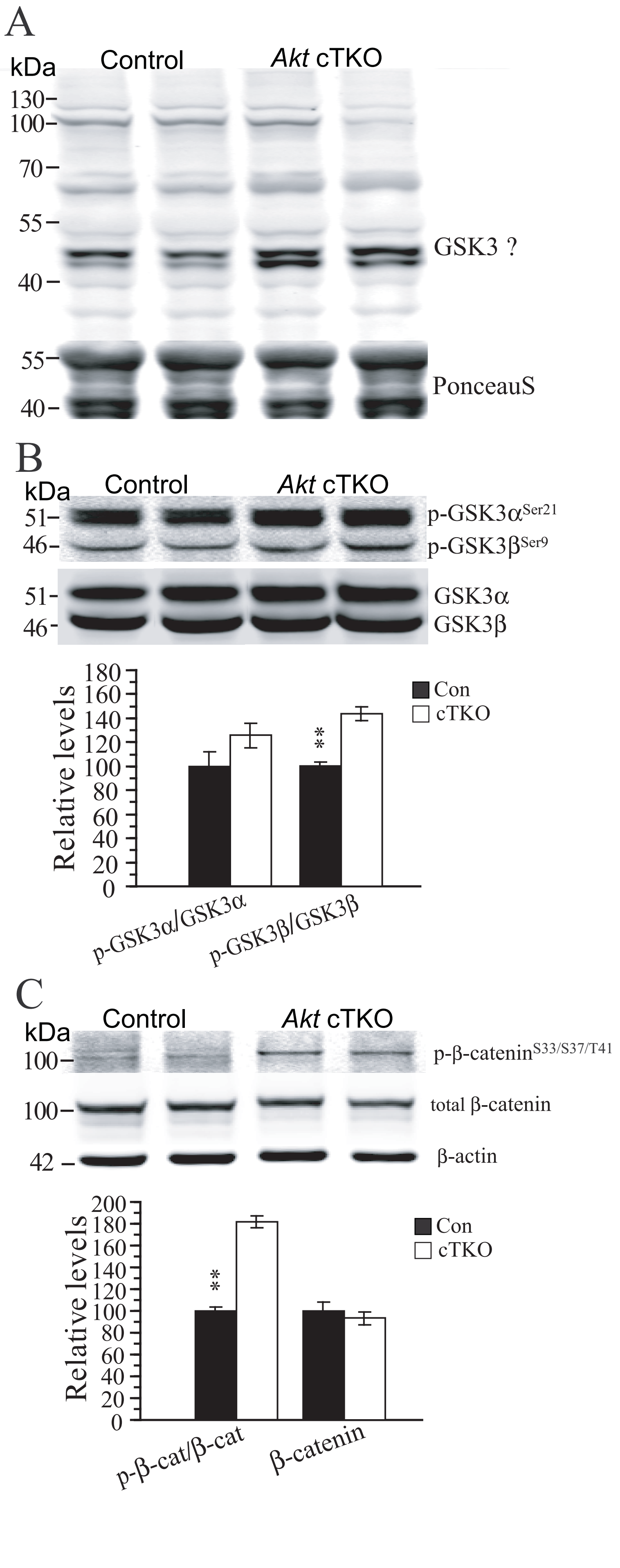
A
kDa
Control
Akt cTKO
130
100
70
55
40
GSK3 ?
55
40
PonceauS
B
kDa
Control
Akt cTKO
51
46
p-GSK3α Ser21
p-GSK3β Ser9
51
46
GSK3α
GSK3β
Relative levels
180
160
140
120
100
80
60
40
20
0
Con
cTKO
**
p-GSK3α/GSK3α
p-GSK3β/GSK3β
C
kDa
Control
Akt cTKO
100
p-β-catenin S33/S37/T41
100
total β-catenin
42
β-actin
Relative levels
200
180
160
140
120
100
80
60
40
20
0
Con
cTKO
**
p-β-cat/β-cat
β-catenin

Supplementary Figure 7. **Biochemical analyses on phosphorylated Akt substrates, p-GSK3α/3β and p-β–catenin in the cortex of *Akt* cTKO mice**. (**A**)An antibody against phosphorylated Akt substrates was used to conduct Western blotting. There were increased intensities on bands with MW of 40 to 70 kDa in *Akt* cTKO mice. Ponceau S was used to stain the membrane and showed comparable levels of loaded proteins for each lane. The control mice were $Akt1^{f/f};Akt2^{+/+};Akt3^{+/+}$ and $Akt1^{f/+};Akt2^{+/+};Akt3^{+/+}$. (**B**)Western blotting on total GSK3α/3β and p-GSK3α^{Ser21}/3β^{Ser9}. There was a small increase on levels of p-GSK3α^{Ser21} (control=100±11.7%, cTKO=125.8±10.3%, $p>0.1$) but a significant increase on levels of p-GSK3β^{Ser9} (control=100±2.9%, cTKO=143.1±5.8%, $p<0.01$) in *Akt* cTKO mice. Relative levels of total GSK3α/3β were not different between control and *Akt* cTKO mice. (**C**)Biochemical analyses on total β-catenin and p-β-catenin. β-catenin is a substrate of GSK3. There were significantly increased levels of p-β-catenin$^{Ser33/Ser37/Thr41}$ (control=100±4.0%, cTKO=181.9±5.8%, $p<0.01$) in *Akt* cTKO mice. Relative levels of total β-catenin (control=100±8.3%, cTKO=93.2±6.2%, $p>0.5$) were not different. β-actin served as the internal loading control.
